# Natural Sunlight Driven Photocatalytic Degradation of Methylene Blue and Rhodamine B over Nanocrystalline Zn_2_SnO_4_/SnO_2_

**DOI:** 10.3390/nano15141138

**Published:** 2025-07-21

**Authors:** Maria Vesna Nikolic, Zorka Z. Vasiljevic, Milena Dimitrijevic, Nadezda Radmilovic, Jelena Vujancevic, Marija Tanovic, Nenad B. Tadic

**Affiliations:** 1 Institute for Multidisciplinary Research, University of Belgrade, 11030 Belgrade, Serbia; zorkav@imsi.bg.ac.rs (Z.Z.V.); milena.dimitrijevic@imsi.bg.ac.rs (M.D.); mtanovic@imsi.bg.ac.rs (M.T.); 2 Institute of Nuclear Sciences of Vinca, National Institute of the Republic of Serbia, University of Belgrade, 11351 Belgrade, Serbia; nadezdas@vin.bg.ac.rs; 3 Institute of Technical Sciences of SASA, 11000 Belgrade, Serbia; jelena.vujancevic@itn.sanu.ac.rs; 4 Faculty of Physics, University of Belgrade, 11000 Belgrade, Serbia; nenad.tadic@ff.bg.ac.rs

**Keywords:** Zn_2_SnO_4_, SnO_2_, heterojunction, photocatalysis, methylene blue, Rhodamine B, natural sunlight

## Abstract

The natural sunlight driven photocatalytic degradation of organic pollutants is a sustainable solution for water purification. The use of heterojunction nanocomposites in this process shows promise for improved photodegradation efficiency. In this work, nanocrystalline Zn_2_SnO_4_/SnO_2_ obtained by the solid-state synthesis method was tested as a heterojunction photocatalyst material for the degradation of methylene blue (MB) and Rhodamine B (RhB) dyes as single and multicomponent systems in natural sunlight. Characterization of the structure and morphology of the synthesized nanocomposite using X-ray diffraction (XRD), Fourier transform infrared spectroscopy (FTIR), field emission scanning electron microscopy (FESEM), transmission electron microscopy (TEM) combined with energy dispersive X-ray spectroscopy (EDS), and photoluminescence (PL) spectroscopy confirmed the formation of Zn_2_SnO_4_/SnO_2_ and heterojunctions between Zn_2_SnO_4_ and the SnO_2_ nanoparticles. A photodegradation efficiency of 99.1% was achieved in 120 min with 50 mg of the photocatalyst for the degradation of MB and 70.6% for the degradation of RhB under the same conditions. In the multicomponent system, the degradation efficiency of 97.9% for MB and 53.2% for RhB was obtained with only 15 mg of the photocatalyst. The degradation of MB occurred through N-demethylation and the formation of azure intermediates and degradation of RhB occurred through sequential deethylation and fragmentation of the xanthene ring, both in single and multicomponent systems.

## 1. Introduction

Photocatalysis, an advanced oxidation process involving the photocatalytic degradation of pollutants in waste water into harmless products such as CO_2_ and water has been the focus of much scientific research due to the continuous and increasing pollution of water and the demand for a clean water supply [1,2]. Organic dyes, applied for coloring in different industrial processes, are a common pollutant [3,4]. The photocatalysis process involves a chemical reaction accelerated by light in the presence of a catalyst material [2]. Photocatalysis degrades organic pollutants into water and carbon dioxide as well as other inorganic substances that are commonly regarded as safe [4].

The photocatalysis process depends on the catalyst material used and the light source. Semiconducting metal oxides have been widely investigated as catalysts in photocatalysis due to their suitable band gap, abundance, versatility, non-toxicity, stability, and photocatalytic activity [5]. Metal oxides commonly investigated and applied for the photocatalytic degradation of organic dyes include TiO_2_ [1] and ZnO [6]. When the surface of a semiconducting catalyst material dispersed in polluted waste water is illuminated with a light source with an energy equivalent or higher than the band gap energy of the semiconducting metal oxide catalyst, this leads to the activation of electrons from the valence band (VB) and their migration to the conduction band (CB) [2]. Positive holes remaining in the VB react with water molecules to form hydroxyl radicals. These radicals degrade the molecules of the organic dye pollutants into intermediates, and in some cases, into carbon dioxide (CO_2_) and water (H_2_O). Excited electrons in the VB can react with dissolved oxygen species, leading to the formation of superoxide radicals that also decompose pollutant species adsorbed on the catalyst semiconductor into intermediate products. Over the years, research has focused on improving the photocatalytic efficiency of metal oxide catalyst materials by modifying them with dopants, forming composites and heterostructures [3,5,7]. Thus, ZnO has been combined with SnO_2_, which showed an improved degradation efficiency of the methylene blue (MB) and Rhodamine B (RhB) organic dyes under UV light [8]. Semiconductor heterojunction catalysis can combine semiconductors with other semiconductors, metals, carbon, or form multicomponent heterojunctions in order to improve the photocatalytic performance, especially in the visible light region [9]. Semiconductor-semiconductor heterojunctions, as heterostructure catalysts, represent a combination of two metal semiconductors with different properties (including ionization potential and electron affinity) [10,11]. When a heterostructure is irradiated by light, electrons are excited from the VBs to CBs of both catalysts. Photogenerated electrons are then injected from the catalyst with a higher CB edge to the catalyst with the lower CB edge, with simultaneous hole injections in the opposite direction, leading to increased charge separation and reduced electron-hole pair recombination [11]. Thus, ZnO/SnO_2_ heterostructures treated by mechanical milling showed an improved photocatalytic degradation of MB in both UV and natural sunlight due to an increase in surface defects and their synergy with heterojunction ZnO/SnO_2_ particles [12]. ZnO/Zn_2_SnO_4_ nanocomposites have shown improved photodegradation efficiency of MB and RhB in visible light, as the synergistic effect of the ZnO and Zn_2_SnO_4_ resulted in an improved lifetime of the photogenerated charge carriers and recombination reduction in photogenerated electron-hole pairs [13].

Wide band-gap semiconductors such as ZnO, SnO_2_, and TiO_2_ have shown good photocatalytic activity in ultraviolet (UV) light irradiation [2]. However, recent research has focused on achieving good photocatalytic properties in visible light, which represents a higher percentage of the solar spectrum. Research has also focused on applying metal oxide nanostructures for the photocatalysis of organic dyes such as MB in natural sunlight [12,14].

Zinc-tin-oxide is an environmentally friendly multicomponent oxide that can have two forms: rhombohedral perovskite ZnSnO_3_ and n-type inverse spinel orthorhombic Zn_2_SnO_4_ [15]. Zn_2_SnO_4_ has high electron mobility, good stability, and a wide band gap, but a relatively unfavorable recombination rate for achieving efficient photocatalysis [16]. The composition, structure and morphology have an influence on the resulting properties of zinc tin oxides, including photocatalytic degradation of organic polluting dyes such as MB and RB [17]. Thus, high porosity was achieved in Zn_2_SnO_4_ synthesized with the aid of different carbon-based templates achieving 96% degradation of MB in 25 min [18]. The optical band gap value of zinc tin oxides depends on the particle size and morphology of the synthesized particles. An interesting feature is that photoluminescence studies of zinc tin oxides have shown the presence of energy levels that can be associated with oxygen vacancies. These sub-gap states enable absorption of photons whose energy is lower than the measured optical band gap and enable photocatalytic activity in visible light besides photocatalytic activity in the UV light region. Urbach energies have been determined for different zinc tin oxide morphologies and compositions, and they also infer possible absorption below the optical band gap [17].

Both Zn_2_SnO_4_ and SnO_2_ are wide band gap semiconductors. An improved photocatalytic degradation of MB in UV light was achieved by Zn_2_SnO_4_/SnO_2_ nanocomposites obtained by sonochemical synthesis combined with high-temperature calcination [19]. The CB edge of Zn_2_SnO_4_ is more negative than the CB edge of SnO_2_, making SnO_2_ a better electron acceptor. At the same time, the VB edge of SnO_2_ is more positive than the VB edge of Zn_2_SnO_4_, leading to a more effective separation of photogenerated electron-holes and a smaller electron-hole recombination [20]. A one-pot hydrothermal method applied for the synthesis of the Zn_2_SnO_4_/SnO_x_ composite achieved improved photocatalytic degradation of methyl orange (MO) organic dye under UV light irradiation [21], while a hydrothermally synthesized Zn_2_SnO_4_/SnO_2_ nanocomposite achieved an enhanced and stable photocatalytic degradation of RhB under UV light irradiation [22]. The molar ratio of Zn:Sn also influenced the resulting photocatalytic activity toward MB degradation under UV light irradiation [23]. Different composite Zn_2_SnO_4_/SnO_2_ microstructures obtained using varied synthesis procedures have also been analyzed and include multi-shelled and hybrid structures [24] as well as hollow engineered microboxes [25], achieving an improved photocatalytic degradation of RhB under simulated sunlight. Hollow cubes of Zn_2_SnO_4_/SnO_2_ showed a better photocatalytic degradation of MB and MO than solid ones in simulated sunlight [26].

The aim of this work was to analyze the use of nanocrystalline Zn_2_SnO_4_/SnO_2_ obtained by a simple method of solid-state synthesis as a heterojunction photocatalyst material for the degradation of single and multicomponent systems of organic dyes in an aqueous medium in natural sunlight. Multicomponent systems containing a mixture of dyes or other active components may have different effects than single-component systems [8] and can show the competitive effects of different dyes in the mixtures [27]. The analysis of these differences is significant from the viewpoint of wastewater treatment processes in the textile industry, especially under natural sunlight [28]. The purpose was to analyze and elucidate the dye decomposition process and degradation mechanism in single-component systems of MB and RhB as well as the interaction of degradation and nanoparticle components in a multicomponent system of MB and RhB organic dyes under the simplest conditions of natural sunlight.

## 2. Materials and Methods

Nanocrystalline Zn_2_SnO_4_/SnO_2_ was obtained by the solid-state synthesis of starting ZnO and SnO_2_ nanopowders (both from Sigma Aldrich, St. Louis, MO, USA, particle size < 100 nm) consisting of mixing/homogenizing the two powders in a 1:1 M ratio with an agate mortar and pestle. Subsequent calcination was performed at 1050 °C for 5 h in a chamber furnace in air, followed by grinding with an agate mortar and pestle. This calcination temperature was selected to ensure Zn_2_SnO_4_ formation in line with previous TG/DTA analysis [29].

The structure of the obtained Zn_2_SnO_4_/SnO_2_ nanocrystalline composite powder was analyzed using X-ray diffraction (XRD) spectroscopy on a Rigaku Ultima IV diffractometer (Tokyo, Japan) in the range of 10–90° with a step of 0.02° and Fourier transform infrared (FTIR) spectroscopy measured on a Perkin Elmer Spectrum Two (Waltham, MA, USA) in the range of 400–4000 cm^−1^ with a resolution of 8 cm^−1^. A Shimadzu UV-2600 device with an ISR2600 Plus Integrating sphere attachment (Kyoto, Japan) was used to measure the UV–Vis diffuse reflectance spectrum. The morphology was observed by field emission scanning electron microscopy (FESEM) on a Tescan MIRA3 XM (Brno, Czech Republic) and transmission electron microscopy (TEM) on an FEI Talos F200X microscope (Thermo Fischer Scientific, Waltham, MA, USA) combined with an energy dispersive X-ray spectroscopy (EDS) system. 3D and 2D photoluminescence (or fluorescence) spectra were acquired using a Fluorolog FL3-221 with a 450 mW Xe lamp (Jobin Yvon Horiba, Paris, France) and FluorEssence 3.5 software (Horiba Scientific, Kyoto, Japan) with the following settings for 3D: excitation range, 325–350 nm; emission range, 446–640 nm; increment 4 nm; slit (band pass) 2 nm for establishing excitation and emission spectra, and 2D spectra settings with an increment of 2 nm for acquiring an emission range of 350–460 nm (400–700 nm) for the excitation of 325 nm (385 nm). The emission detector signal was scaled by the reference quantum counter signal (S1/R1).

The photocatalytic activity of Zn_2_SnO_4_/SnO_2_ was evaluated by monitoring the photodegradation of aqueous solutions of methylene blue (MB) and Rhodamine B (RhB) individually and combined in binary dye mixtures under direct natural sunlight in July and August 2022 between 11.00 a.m. and 01.00 p.m. (average daily temperature 30 ± 2 °C). The experiments were conducted on fully sunny days with no clouds, and the light intensity was measured as 800–1000 W/m^2^ using a Voltcraft PL-110SM solar radiation measuring instrument. We varied the photocatalyst amount from 10 to 50 mg and applied it to 50 mL 10 ppm aqueous solutions of MB, RhB, or a 50 mL equimolar mixture of 10 ppm MB and RhB. Before irradiation, the solutions were mixed in the dark on a magnetic mixer for 60 min to achieve adsorption and desorption equilibrium between the photocatalyst and pollutant. Photolysis of the solutions was also analyzed under the same conditions. Absorption was monitored on a Shimadzu UV-2600 spectrophotometer (Kyoto, Japan) at 663 nm (MB) and 554 nm (RhB).

The degradation efficiency was determined as:

(1) $$\%\;\mathrm{degradation}\;\mathrm{efficiency}=\frac{\left(C_{0}-C_{t}\right)}{C_{0}}\times 100$$

where *C*_0_ is the concentration of the dye solution at the start of the exposure to natural sunlight (*t* = 0) and *C_t_* is the concentration of the dye solution at time *t*.

Kinetic analysis of the dye degradation process was performed using the simplified Langmuir–Hinshelwood (L-H) kinetic model [14,24,27]:

(2) $$-\ln\frac{C_{t}}{C_{0}}=-\ln\frac{A_{t}}{A_{0}}=kt$$

where the normalized temporal concentration changes of the dye during the photocatalytic process can be assumed to be proportional to the normalized maximum absorbance change of the dye at time *t* (*A_t_*) and 0 (*A*_0_) derived from the change in the absorption profile at irradiation time *t*. The pseudo-first-order kinetic constant (*k*) can be determined from the fitted linear relation.

Mass spectrometry (MS) analysis was performed on a TSQ Quantum Access Max mass spectrometer equipped with a HESI source (Thermo Fisher Scientific, Waltham, MA, USA). The ion source settings were as follows: spray voltage, 3500 V; vaporizer temperature, 300 °C; sheath gas, N_2_; pressure, 27 AU; ion sweep gas pressure, 1.0 AU; auxiliary gas (N_2_) pressure, 10 AU; capillary temperature, 275 °C; skimmer offset, 0 V. Data were acquired in positive mode including full scanning in the m/z range from 100 to 1000 for qualitative analysis and product ion scanning mode for the quantitative analysis. Collision-induced fragmentation experiments were performed using Ar as the collision gas, with the collision energy set at 10 eV. Samples were introduced into the mass spectrometer with a syringe pump and continuous flow injection at a flow rate of 10 μL min^−1^ for a period of 3 min. Xcalibur software Analyst version 1.4 (Thermo Fisher Scientific) was used for data acquisition and processing.

## 3. Results and Discussion

### 3.1. Structure, Morphology, and Optical Properties

**Figure 1:**
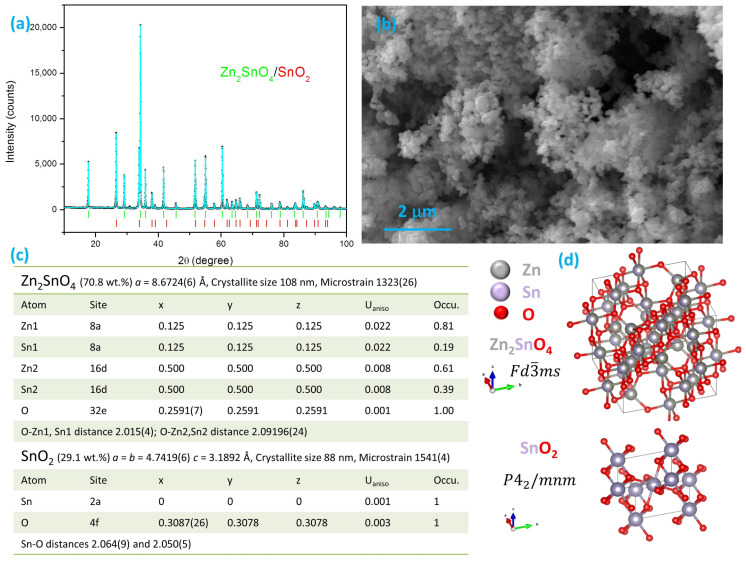
XRD pattern (points) measured for the Zn_2_SnO_4_/SnO_2_ powder and Rietveld refinement (pale blue line) with the identified phases marked in green—Zn_2_SnO_4_ and red—SnO_2_ (**a**), FESEM image of the Zn_2_SnO_4_/SnO_2_ powder (**b**), structural parameters determined from the Rietveld refinement (**c**), and crystal structure drawn by VESTA [31] for Zn_2_SnO_4_ and SnO_2_ (**d**).

The measured XRD pattern of the synthesized Zn_2_SnO_4_/SnO_2_ powder is shown in Figure 1a and confirms the formation of two phases: cubic spinel Zn_2_SnO_4_ (JCPDS 24-1470) and tetragonal SnO_2_ (JCPDS 88-0287). The FESEM image of the Zn_2_SnO_4_/SnO_2_ powder (Figure 1b) showed relatively uniform nanoparticles of Zn_2_SnO_4_ and SnO_2_ with a similar shape and size. The average particle size was estimated as ~150 nm. Rietveld refinement of the measured XRD pattern using the GSAS II software package [30] showed good agreement between the measured and refined patterns (Figure 1a) and enabled the determination of structural parameters (unit cell parameters, atomic positions, crystallite size, and microstrain), as shown in Figure 1c. The crystal structures drawn using the determined crystalline lattice parameters for Zn_2_SnO_4_ ($Fd\bar{3}ms$) and SnO_2_ (*P*4_2_/*mnm*) using the VESTA software package [31] are shown in Figure 1d. The Zn ions are shown in pale grey and the Sn ions are pale purple, while the oxygen ions are shown as red balls. For Zn_2_SnO_4_, we obtained the inversion parameter of 0.81, which is in the range previously determined for zinc stannate [29,32,33] and confirms the presence of randomly distributed Zn^2+^ and Sn^4+^ ions on both tetrahedral and octahedral sites in the cubic spinel lattice (as shown in Figure 1d). The ideal cation symmetry for the cubic spinel space groups of Zn_2_SnO_4_ was distorted, as the oxygen parameter was determined to be 0.2591 (rather than 0.25), as noted previously for zinc stannate with larger tetrahedra and smaller octahedra [29]. The crystallite sizes of 108 nm for Zn_2_SnO_4_ and 88 nm for SnO_2_ were similar to values previously obtained for this composite [29].

**Figure 2:**
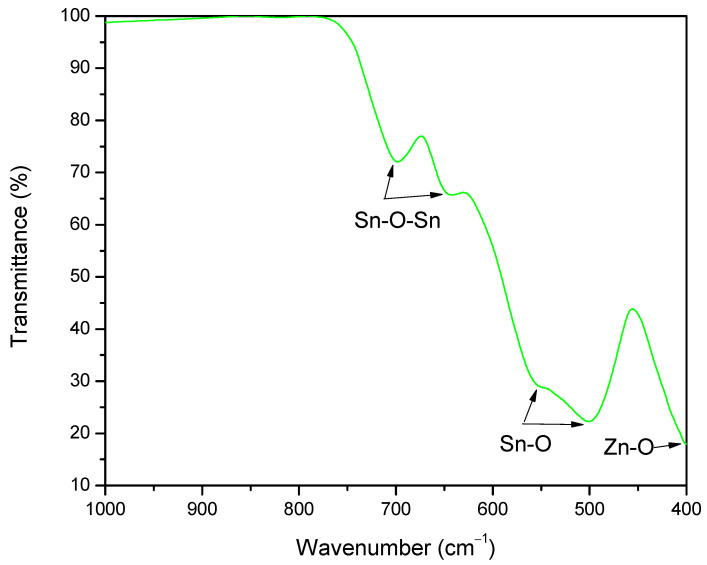
FTIR spectrum measured for the Zn_2_SnO_4_/SnO_2_ powder.

The measured FTIR spectrum of Zn_2_SnO_4_/SnO_2_ shown in Figure 2 confirms the presence of metal-oxide bonds in the nanocomposite. Thus, Sn−O stretching vibrations can be noted in the range 430–620 cm^−1^ (501 and 553 cm^−1^), and Sn−O−Sn band vibrations were present in the range 645–700 cm^−1^ (645 and 698 cm^−1^), as noted before for Sn present in both SnO_2_ and Zn_2_SnO_4_ [29]. Zn-O vibration bands were anticipated in the range 150–420 cm^−1^, and the tail of one peak was possibly present (marked in Figure 2), as previously noted [29].

**Figure 3:**
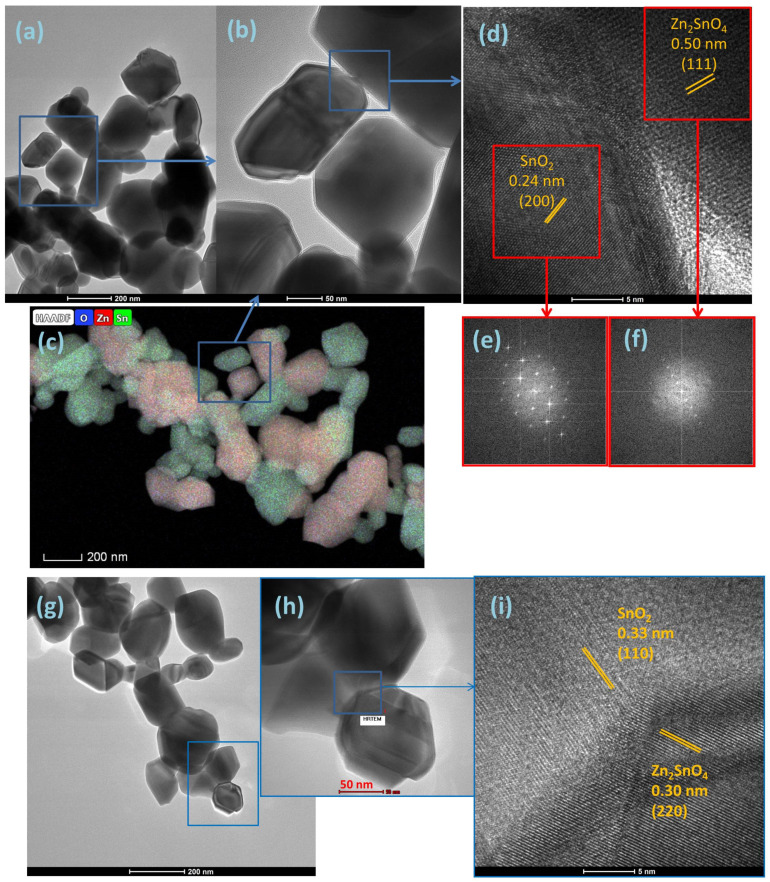
TEM (**a**,**b**,**g**,**h**), HAADF TEM (**c**), HRTEM (**d**,**i**), and FFT (**e**,**f**) images for the Zn_2_SnO_4_/SnO_2_ powder; blue rectangles mark the magnified areas, while the red squares mark the areas used for FFT analysis.

The morphology and structure of the Zn_2_SnO_4_/SnO_2_ powder were further analyzed by TEM, high-resolution TEM (HRTEM), high-angle annular dark-field scanning (HAADF) TEM, and EDS. The TEM images shown in Figure 3a,b enabled us to have a closer look at the powder particles, confirming that the SnO_2_ and Zn_2_SnO_4_ nanoparticles were similar in size and shape. HAADF combined with EDS mapping, as shown in Figure 3c, further confirmed the presence of a homogenous mixture of the two components of the nanocomposite powder. Analysis of the periodic lattice fringes for the two particles shown in the HRTEM image in Figure 3d using fast Fourier transform (FFT) enabled us to determine a lattice spacing of 0.24 nm for the (200) plane of tetragonal SnO_2_ and 0.50 nm for the (111) plane of cubic spinel Zn_2_SnO_4_, with corresponding FFT images for SnO_2_ (Figure 3e) and Zn_2_SnO_4_ (Figure 3f). Further analysis of the TEM images of Zn_2_SnO_4_/SnO_2_, as shown in Figure 3g,h, and the HRTEM image shown in Figure 3i, revealed a lattice spacing of 0.33 for the (110) plane of tetragonal SnO_2_ and a lattice spacing of 0.30 for the (220) plane of cubic spinel Zn_2_SnO_4_. The SnO_2_ plane and the neighboring Zn_2_SnO_4_ plane formed a heterointerface with no interlayer gaps between them, which indicated the presence of Zn_2_SnO_4_ and SnO_2_ heterojunctions, as noted before for Zn_2_SnO_4_/SnO_2_ microcubes [34]. Combining two individual oxides in a nanocomposite that forms heterojunctions between SnO_2_ and Zn_2_SnO_4_ has previously been noted for this type of nanocomposite, which has led to improved sensing and photocatalytic properties [35,36,37]. The close proximity and increased contact between Zn_2_SnO_4_ and SnO_2_ particles in the synthesized heterostructures resulted in the formation of heterojunctions in the interfacial region and led to faster electron transfer [38,39].

Generally, the surface area of materials obtained by solid-state synthesis is not very high, and this was confirmed by the BET surface area determined from the measured nitrogen adsorption/desorption isotherms (type II) of 4.9 m^2^/g (Zn_2_SnO_4_/SnO_2_ obtained by solid-state synthesis with a shorter calcination time) [29]. This value was slightly lower but comparable with some previous results obtained for this composite such as 9.9 m^2^/g obtained for microcubes [34] or 13.6 m^2^/g obtained for a specific cube-type morphology [20]. The mercury porosimetry measurements in [29] of the solid-state obtained Zn_2_SnO_4_/SnO_2_ indicated a stable pore system with high porosity values obtained for both intrusion/extrusion runs. The average pore diameters were similar and close to the average particle size. Taking into account the results obtained by the nitrogen adsorption/desorption measurements and the mercury porosimetry measurements, we concluded that the analyzed nanocomposite was macroporous with very little micro and mesopores, similar to that expected for the Zn_2_SnO_4_/SnO_2_ powder synthesized in this work. Even though a high BET specific surface area was not obtained by solid-state synthesis, the macroporous microstructure is also beneficial for photocatalysis.

**Figure 4:**
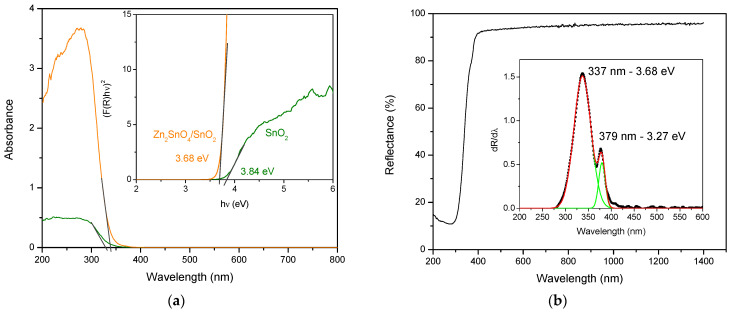
Zn_2_SnO_4_/SnO_2_ (orange) and SnO_2_ (green) powder absorbance obtained by Kubelka–Munk approximation (**a**) from the measured diffuse reflectance spectra (**b**), insets (**a**)—Tauc plot for estimation of direct band gap and (**b**)—first derivative of the measured diffuse reflectance spectra of Zn_2_SnO_4_/SnO_2_ (black points) and the calculated transition energy values (the red line represents the sum of fitted peaks, while the green lines represent the Gaussian fits of the two noted peaks with maxima at 337 and 379 nm, respectively).

Kubelka–Munk transformation of the measured diffuse reflectance spectrum of the Zn_2_SnO_4_/SnO_2_ powder enabled the calculation of the equivalent absorption coefficient used for the estimation of the optical band gap, as shown in Figure 4 [40]. The direct band gap of the analyzed Zn_2_SnO_4_/SnO_2_ powder was estimated from the Tauc plot to be 3.87 eV (inset in Figure 4a). A direct band gap was assumed, taking into account that both SnO_2_ and Zn_2_SnO_4_ have direct optical energy gaps. SnO_2_ has a direct optical energy gap estimated to be 3.68 eV [41], though for nanoparticles and thin films, it has been found to be between 3.4 and 4.6 eV [42,43]. Zn_2_SnO_4_ also has a direct optical energy gap, which has been found to be between 3.2 and 4.1 eV, with 3.6 eV determined for bulk samples, but both higher and lower for nanoparticles and films with different morphologies [17,21,44,45]. The absorption edge for Zn_2_SnO_4_/SnO_2_ was estimated to be 337 nm (3.68 eV), as shown in Figure 4a. The estimated direct band gap value from the Tauc plot confirmed this value, as shown in the inset in Figure 4a. The estimated band gap value of 3.68 eV was slightly higher than the value of 3.63 eV determined by Li et al. [20] for a cube-shaped Zn_2_SnO_4_/SnO_2_ heterojunction composite powder. The absorption edge of pure SnO_2_ powder (used in the synthesis) was lower than the absorption edge of Zn_2_SnO_4_/SnO_2_. A redshift in the absorption edge of Zn_2_SnO_4_/SnO_2_ compared with the absorption edge of pure SnO_2_ is an indication of a narrower band gap caused by the formation of heterojunctions between Zn_2_SnO_4_ and SnO_2_, as previously noted [39]. The Tauc plot shown in the inset enabled the estimation of the direct band gap of 3.84 eV. Ye et al. [39] concluded that heterojunction formation between Zn_2_SnO_4_ and SnO_2_ in a heterostructured nanocomposite can be confirmed by a band gap value higher than the band gap of pure Zn_2_SnO_4_ and lower than the band gap of pure SnO_2_. Wang et al. [21] found that heterojunctions formed at the interface between SnO_2_ and Zn_2_SnO_4_ promoted a better separation of photoinduced electrons (e^−^) and holes (h^+^), leading to an extended absorption range and improved photocatalytic activity.

The first derivative of the measured reflectance (d*R*/dλ) has been used to enable the identification of additional transition energies as well as estimate the band gap [40,46]. The presence of additional energy levels in the band gap of doped metal oxides or metal oxide nanocomposites has led to enhanced sensing properties or improved electrocatalytic activity [47,48]. In the case of Zn_2_SnO_4_/SnO_2_, there was one prominent peak at 337 nm accompanied by a smaller but noticeable shoulder at 379 nm, showing the presence of additional transitions within the band gap (Figure 4b).

**Figure 5:**
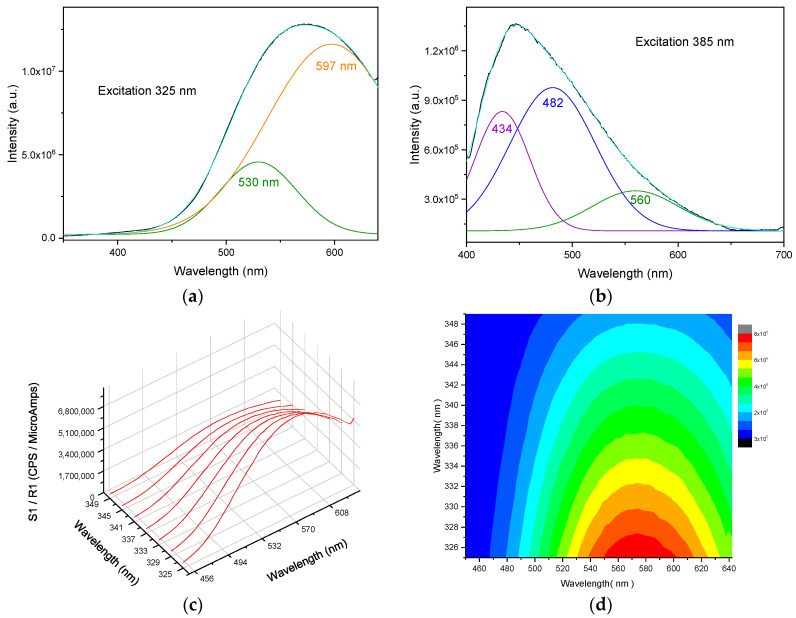
PL spectra of Zn_2_SnO_4_/SnO_2_ with an excitation wavelength of 325 nm (**a**) and 385 nm (**b**) (the black points represent the measured spectra, the pale blue lines represent the sums of fitted peaks, and the green, orange, purple and blue lines represent the Gaussian fits of the individual peaks). 3D PL spectrum (**c**) and contour plot (**d**) under an excitation range of 325–350 nm and emission range of 446–640 nm.

The photoluminescence (PL) spectra of Zn_2_SnO_4_/SnO_2_ measured under an excitation of 325 and 385 nm are shown in Figure 5a,b, while the photoluminescence 3D spectrum of Zn_2_SnO_4_/SnO_2_ measured in the emission range of 446–640 nm for the excitation range of 325–350 nm is shown in Figure 5c, and the contour plot of this spectrum is shown in Figure 5d. PL emission spectra provide information on the migration and recombination of photo-induced hole-electron pairs [16]. The emission intensity decreased with an increase in excitation wavelength, as shown in Figure 5c,d with a maximum intensity at around 575 nm for an excitation of 325 nm. After multi-peak Gaussian fitting, the spectrum obtained under an excitation of 325 nm (Figure 5a) was deconvoluted into a green and an orange emission band located at 530 nm (2.33 eV) and 597 nm (2.07 eV), respectively. The spectrum obtained under the excitation of 385 eV (Figure 5b) was deconvoluted into a violet, blue, and green emission band located at 434 nm (2.85 eV), 482 (2.57 eV), and 560 nm (2.21 eV), respectively. Similar emission bands have previously been observed in Zn_2_SnO_4_/SnO_2_ nanocomposites [22] as well as in Zn_2_SnO_4_ [49] and SnO_2_ [50]. The determined emission bands can be ascribed to different luminescent centers due to defect energy levels originating from oxygen vacancies, tin vacancies, or oxygen interstitials [13,49,50].

### 3.2. Photodegradation of Dye in Natural Sunlight

#### 3.2.1. Photodegradation of MB

**Figure 6:**
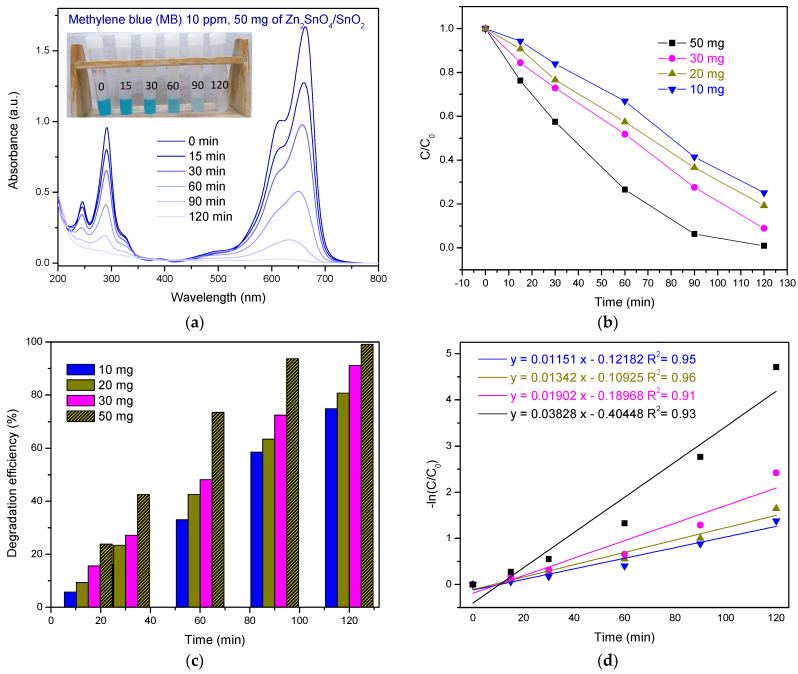
UV–Vis absorption spectra of the photocatalytic degradation of MB (10 ppm) under natural sunlight irradiation in the presence of 50 mg of Zn_2_SnO_4_/SnO_2_ (**a**), C/C_0_ at different time intervals (**b**), degradation efficiency (**c**), and the pseudo-first-order kinetic model (**d**) for different photocatalyst loads (10, 20, 30, and 50 mg).

Figure 6a shows that the degradation of MB dye (10 ppm = 10 mg/L) under natural sunlight irradiation using 50 mg of the photocatalyst resulted in a noticeable and significant reduction in the absorption peaks of MB and in an almost white solution (as shown in the inset), indicating the almost complete degradation of the dye after 120 min (2 h) of irradiation in natural sunlight. MB is a blue cationic thiazine dye with absorption peaks at 246 nm, 291 nm, and 663 nm and a shoulder at 615 nm, three mesomeric structures where the positive charge is located either on nitrogen or sulfur atoms [51]. The reduced forms (Leuco-methylene blue and MBH_2_^+^) obtained using reducing agents are colorless and stable in aqueous solutions. With an increase in the irradiation time, we also noted a slight blueshift in the strongest absorption band starting at 663 nm, which reduced to 636 nm after 120 min. The occurrence of a blueshift has been previously noted for photocatalytic MB degradation with other photocatalysts such as PANI/TiO_2_ composites [52]. This is associated with the occurrence of N-demethylated derivatives of MB during the degradation process. Variation in the Zn_2_SnO_4_/SnO_2_ concentration from 10 to 50 mg (Figure 6b) showed that a decrease in the number of active sites on the catalyst surface reduced the photocatalytic activity and the characteristic absorption peak at 663 nm. The blueshift remained, but was less with a decrease in catalyst load (650 nm–10 mg, 649 nm–20 mg, and 638 nm–50 mg). The degradation efficiency of 99.1% was achieved after 120 min of irradiation in natural sunlight for 50 mg of the photocatalyst, which was reduced to 91.1% for 30 mg, 80.8% for 20 mg, and 74.8% for 10 mg of the photocatalyst, as shown in Figure 6c. The photodegradation rate followed the pseudo-first-order kinetic model (Equation (2)) as shown in Figure 6d, with R^2^ values between 0.91 and 0.96. The determined pseudo-first-order kinetic constant k values showed an increase with the increase in catalyst load, with 0.01151 for 10 mg and 0.03828 for 50 mg.

Methylene blue (MB) degradation has been previously achieved by this type of composite, but mostly in UV light, as shown in Table 1, and shows a significant influence of the composite morphology on the degradation efficiency and pseudo-kinetic constant. Rovisco et al. [17] also showed that the morphology and phase had a significant influence on the photocatalytic activity of zinc-stannate in both UV and visible light for the degradation of MB. Li et al. [26] attributed the improved photocatalytic activity of Zn_2_SnO_4_/SnO_2_ hollow cubes compared with solid ones to the presence of cavities within the cubes, enabling higher light absorption, a lower recombination rate of photogenerated electrons, and a highly efficient separation of photoelectrons from vacancies. In this case, the presence of heterojunctions between Zn_2_SnO_4_ and SnO_2_ nanoparticles can bring about improved photocatalytic activity due to the improved separation of photoinduced electrons and holes [21]. This was reflected in the high value obtained for the pseudo-kinetic constant for 50 mg of photocatalyst as well as the relatively high values for lower catalyst loads, showing that the complete degradation of MB would occur for durations longer than 120 min, but comparable with the results shown in Table 1.

#### 3.2.2. Photodegradation of RhB

**Figure 7:**
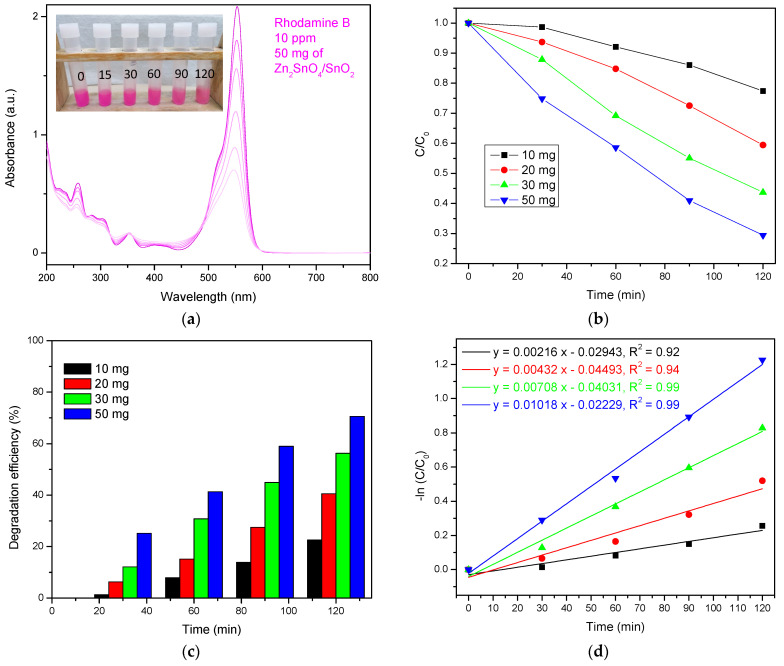
UV–Vis absorption spectra of the photocatalytic degradation of RhB (10 ppm) under natural sunlight irradiation in the presence of 50 mg of Zn_2_SnO_4_/SnO_2_ (**a**), C/C_0_ at different time intervals (**b**), degradation efficiency (**c**), and pseudo-first-order kinetic model (**d**) for different photocatalyst loads (10, 20, 30, and 50 mg).

The degradation of RhB in natural sunlight using the Zn_2_SnO_4_/SnO_2_ heterojunction nanocomposite was less efficient than the degradation of MB under the same conditions. Though they are both cationic dyes, MB is a blue cationic thiazine dye, while RhB is a pink dye part of the triphenylmethane family. Figure 7a shows the absorption spectra measured for RhB degradation in natural sunlight using 50 mg of Zn_2_SnO_4_/SnO_2_ as the photocatalyst. Though there was discoloration of the solution, which changed from bright pink to a lighter, more milky pink, as shown in the inset, 120 min was not sufficient for the complete degradation of RhB. We also noted a slight blueshift of the characteristic peak at 554 nm, which shifted to 547 nm after 120 min of natural sunlight irradiation. This blueshift was attributed to the formation of N-deethylated intermediates of RhB and has been previously noted for both the Zn_2_SnO_4_/SnO_2_ nanocomposite [22] and other photocatalysts such as NaBiO_3_ [54]. The photocatalytic activity was lower for smaller catalyst loads, as shown in Figure 7b. The highest degradation efficiency of 70.6% was obtained for the highest catalyst load of 50 mg, and it decreased with a decrease in catalyst load, with 10 mg achieving a degradation efficiency of only 22.6% after 120 min in natural sunlight, as shown in Figure 7c. The photodegradation rate also followed the pseudo-first-order kinetic model (Equation (2)), as shown in Figure 7d, with R^2^ values between 0.92 and 0.99. The determined pseudo-first-order kinetic constant k values increased with the increase in catalyst load, with 0.00216 for 10 mg and 0.01018 for 50 mg. Table 2 shows a comparison between the results obtained in this work for the Zn_2_SnO_4_/SnO_2_ nanocomposite heterojunctions with the literature data obtained for different Zn_2_SnO_4_/SnO_2_ nanocomposite morphologies and light sources (simulated UV or UV–Vis light). The kinetic rate we obtained was not the highest, but also not the lowest, indicating that for a longer irradiation time in natural sunlight, we could expect the complete degradation of RhB, possibly after 240 min.

#### 3.2.3. Photodegradation of MB+RhB

**Figure 8:**
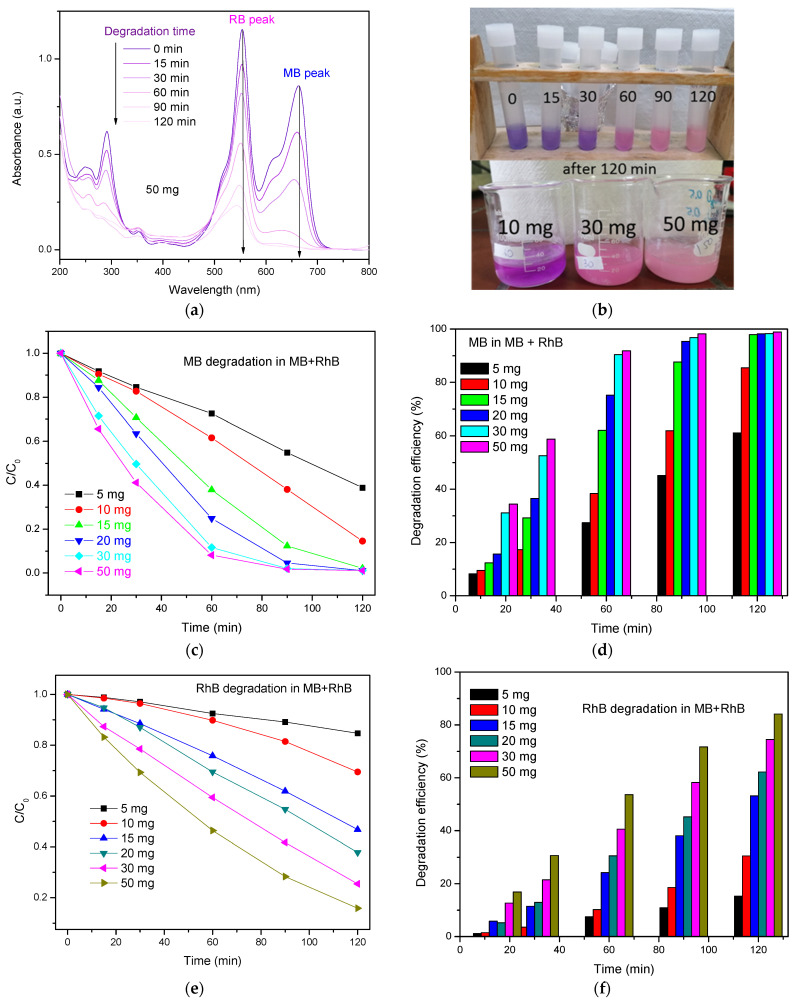

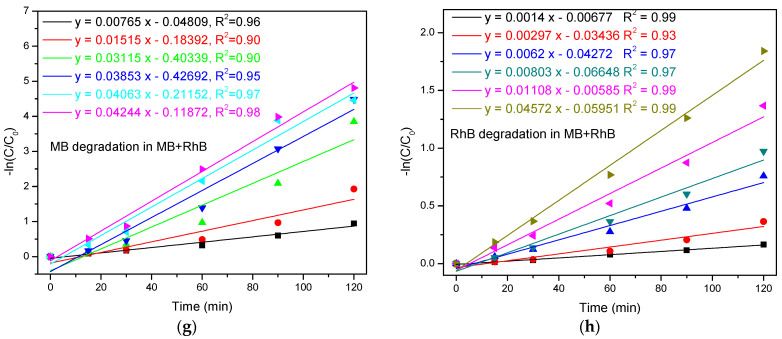
UV–Vis absorption spectra of the photocatalytic degradation of MB+RhB (10 ppm) under natural sunlight irradiation in the presence of 50 mg of Zn_2_SnO_4_/SnO_2_ (**a**), color change in the solution after 120 min using 50 mg of Zn_2_SnO_4_/SnO_2_ and final solutions after irradiation for 120 min using 10, 30, and 50 mg Zn_2_SnO_4_/SnO_2_ (**b**), C/C_0_ at different time intervals for MB (**c**) and RhB (**e**) and degradation efficiency for MB (**d**) and RhB (**f**) in MB+RhB, and the pseudo-first-order kinetic model for MB (**g**) and RhB (**h**) in MB+RhB for different photocatalyst loads (5–50 mg).

The measured absorption curves showing the degradation of MB+RhB under natural sunlight using 50 mg of Zn_2_SnO_4_/SnO_2_ are presented in Figure 8a, indicating the complete degradation of MB and the significant degradation of RhB. A blueshift in both characteristic peaks for MB and RhB (at 663 and 554 nm) was also noted, indicating the presence of both N-demethylated N-deethylated intermediates of MB and RhB, respectively, in the mixed dye solution. Widening of the absorption bands was also noticeable after longer irradiation times. As expected from the results obtained for the degradation of MB and RhB as single dye aqueous solutions, MB degraded faster compared with RhB. The starting purple solution completely changed color after 120 min of irradiation, resulting in a milky pale pink color, as shown in Figure 8b. Change in the catalyst amount (10 and 30 mg) showed a paler purple for 10 mg and a darker shade of pink after 120 min for 30 mg of Zn_2_SnO_4_/SnO_2_. Figure 8c,e, and Figure 8d,f shows the change in dye concentration ratios and the degradation efficiency of MB and RhB in MB+RhB, respectively, with the catalyst load varying between 5 and 50 mg. The degradation efficiency of MB was higher, achieving 97.9% with only 15 mg of photocatalyst. The degradation efficiency of 84.1% was obtained for RhB in MB+RhB with 50 mg of the photocatalyst. Analysis of the first-order reaction kinetics showed that the determined pseudo-first-order kinetic constant k values were overall higher for the same catalyst load applied to the single dye solutions (Figure 8g,h). Thus, 30 mg of Zn_2_SnO_4_/SnO_2_ degraded RhB, and 20 mg of Zn_2_SnO_4_/SnO_2_ degraded MB with a similar kinetic rate to 50 mg of Zn_2_SnO_4_/SnO_2_ in single dye solutions. Thus, we can conclude that Zn_2_SnO_4_/SnO_2_ nanocomposite heterojunctions perform better in a mixed MB+RhB dye solution. These changes could be attributed to adsorption competition, light shielding, or some effects of dye–dye interactions. Dlugosz et al. [8] studied the degradation of single, bi, and multiple mixed dye solutions using the ZnO-SnO_2_ nanocomposite and determined that the presence of components of the same nature did not affect the dye decomposition rate, except for quinolone yellow (dye with a neutral character), while Tang et al. [27] noted a 5% reduction in degradation rate for malachite green (cationic dye). MB and RhB degradation did not change in the mixed dye solutions.

#### 3.2.4. Zn_2_SnO_4_/SnO_2_ Pollutant Degradation Mechanism

**Figure 9:**
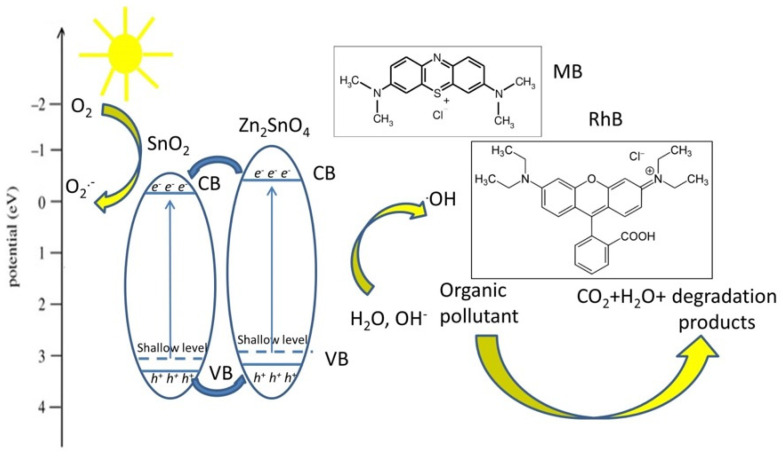
Degradation mechanism scheme for MB, RhB, and MB+RhB degradation under natural sunlight by Zn_2_SnO_4_/SnO_2_.

Irradiation of the Zn_2_SnO_4_/SnO_2_ nanocomposite with natural sunlight initiates electron excitation from valence bands (VBs) to conduction bands (CBs) for both components. Then, the electron (e^−^) transfer occurs from Zn_2_SnO_4_ with a higher CB edge to SnO_2_ with the lower CB edge (as shown in Figure 9). Simultaneous hole (h^+^) transfer also occurs from SnO_2_ to Zn_2_SnO_4_, which leads to more effective charge carrier separation, thus reduced charge carrier recombination increases the carrier lifetime [11,22]. Photogenerated electrons in the CBs can react with O_2_ absorbed on the catalyst (Zn_2_SnO_4_/SnO_2_ nanocomposite) surface or dissolved in water and create superoxide anion radicals (O_2_^−^). At the same time, photogenerated holes can interact with OH^−^ or H_2_O and create hydroxyl radicals (OH·) [22]. Both superoxide anion and hydroxide radicals are responsible for the degradation of organic pollutants (MB, RhB, MB+RhB) into CO_2_ and H_2_O [17]. The proposed degradation mechanism is shown in Figure 9.

### 3.3. MS Analysis—Degradation Mechanism

In order to elucidate the degradation pathways of MB, RhB, and their mixture under photocatalytic conditions in greater detail, an electrospray ionization mass spectrometry (ESI-MS) analysis was carried out in positive mode. The obtained spectra indicated a stepwise degradation process that occurs through the formation of intermediate products and can be classified into three main mechanisms: N-demethylation, ring opening, and complete fragmentation into smaller organic residues. The degradation process was monitored at time intervals of 0, 15, 30, 60, 90, and 120 min, which allowed for an observation of the kinetics and progressive fragmentation of the dye molecules. The results provide deeper insights into the individual stages of degradation, simultaneously confirming the spectral shifts and changes in absorption observed in UV–Vis analysis, thereby further contributing to the understanding of the photocatalytic degradation mechanism of these dyes and their mixture.

#### 3.3.1. MB Degradation Pathway

**Scheme 1:**
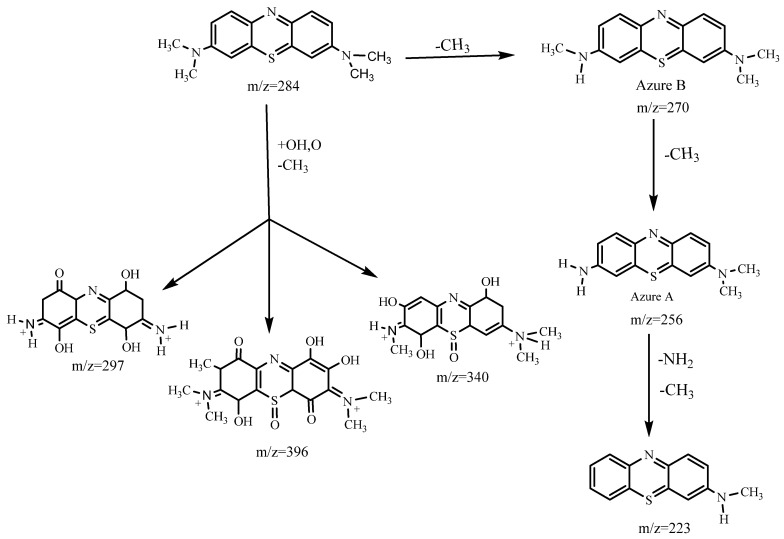
Presumed degradation mechanism of MB during photodegradation in the presence of Zn_2_SnO_4_/SnO_2_.

**Figure 10:**
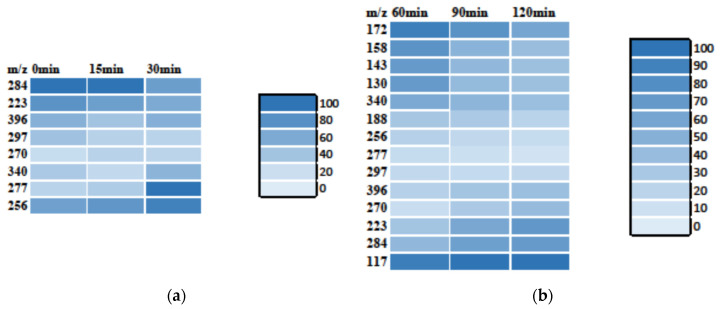
Heatmaps illustrating the change in intensity of the intermediary peaks during MB degradation in the first 30 min (**a**) and the 60–120 min interval (**b**) in the presence of Zn_2_SO_4_/SnO_2_ under natural sunlight.

The ESI-MS spectrum of the initial MB solution (MB 0) showed a dominant molecular ion peak at *m*/*z* 284 [56,57,58,59,60,61], confirming the presence of intact MB. After 15 min of irradiation (MB 15), peaks mainly appeared at *m*/*z* 256 and *m*/*z* 270 corresponding to Azure A, and Azure B, respectively [57]. The peak at *m*/*z* 223 corresponded to the N-demethylation products, as suggested by the observed blueshift in the UV–Vis spectrum (Figure 4). The peak at *m*/*z* 297 can be explained by multiple hydroxylation of the MB molecule [58,60]. The spectrum after 30 min of irradiation did not show any significant changes, but as the irradiation continued (MB 60), the formation of peaks at *m*/*z* 188, 172, and 158 [59] revealed oxidative fragmentation and ring-opening processes, aligning with the diminishing absorption intensity in the UV–Vis measurements. The increase in the intensity of products formed by oxidation and hydroxylation of the MB molecule, particularly in the final stages (MB 90 and MB 120), led to the conclusion that MB had been completely degraded. Scheme 1 presents the presumed degradation mechanism of MB during photodegradation in the presence of Zn_2_SnO_4_/SnO_2_, while the possible structures of degradation products detected at various UV exposure times are provided in Table 3. The heatmaps shown in Figure 10, which track the relative intensities of the peaks in the mass spectra over time (up to 30 min—Figure 10a, and from 60 min onward—Figure 10b), illustrate the variation in the abundance of intermediates during MB photodegradation. These data demonstrate that as the initial intensity of intact MB (*m*/*z* 284) gradually decreases, early intermediates, such as N-demethylation products (*m*/*z* 223), Azure A (*m*/*z* 256), and Azure B (*m*/*z* 270), form rapidly, but their intensity decreases as the reaction progresses, confirming that they are transient degradation products [59]. The intermediate product at *m*/*z* 277 exhibited oscillatory dynamics, suggesting a continuous transformation. The formation of oxidative products, especially the peaks at *m*/*z* 340 and *m*/*z* 396, became more pronounced in the later stages, supporting the hypothesis that MB does not degrade solely via demethylation but also through advanced oxidative transformations that lead to the final fragmentation and eventual mineralization of the initial molecule. However, additional validation is required. The observed evolution of the peak intensities indicates that MB degradation occurs through a sequence of interconnected reactions from the initial demethylation and hydroxylation processes, through the formation of intermediate oxidized products, to complete fragmentation and mineralization.

#### 3.3.2. RhB Degradation Pathway

**Scheme 2:**
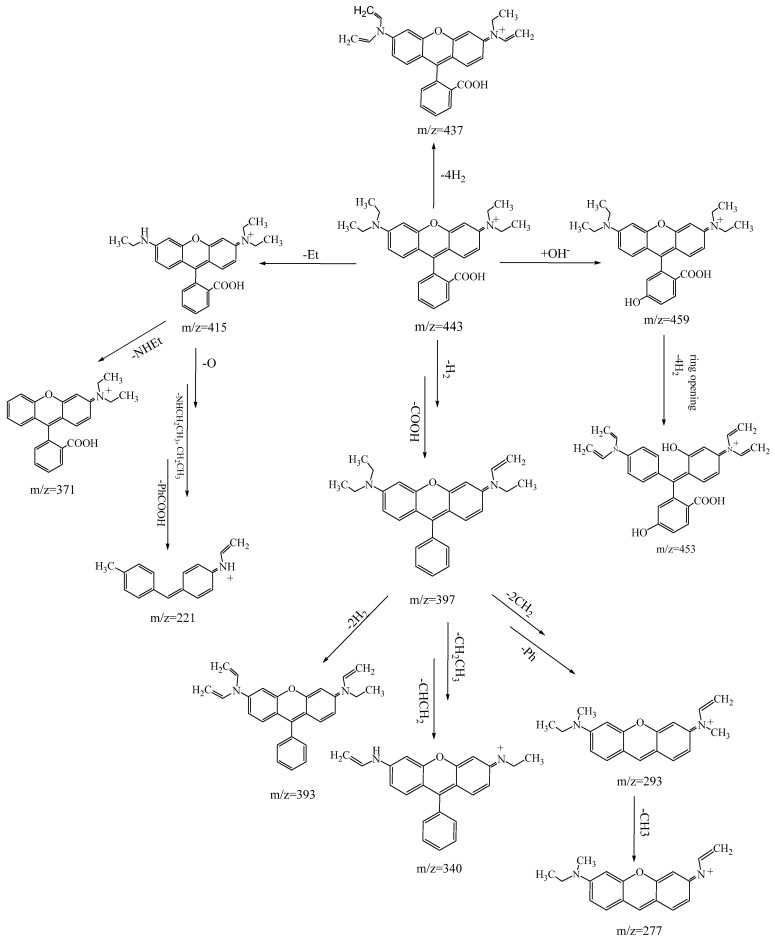
Presumed degradation mechanism of RhB during photodegradation in the presence of Zn_2_SO_4_/SnO_2_.

**Figure 11:**
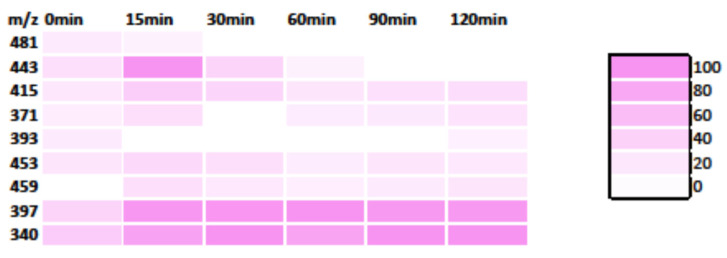
Heatmap illustrating the change in intensity of intermediary peaks during RhB degradation in the presence of Zn_2_SO_4_/SnO_2_ for 0–120 min under natural sunlight.

Based on the heatmap of *m*/*z* intensity values from the MS spectra of RhB photodegradation (time intervals: 0, 15, 30, 60, 90, and 120 min) shown in Figure 11, it is possible to hypothesize a photodegradation pathway for Rhodamine B that essentially consists of two notable processes: sequential N-deethylation [54,61,62,63] and fragmentation of the xanthene ring [54]. In addition to these two mechanisms, dealkylation, deamination, decarboxylation, dehydration, chromophore cleavage or disruption of conjugation, and rupture of the cyclic structure were also observed. The molecular ion (Rhb0) at *m*/*z* 443 [54,61,62,63,64] increased during the first 15 min and then decreased significantly by 60 min, eventually being present at less than approximately 6%. This trend indicates that the main molecular ion of RhB is rapidly consumed, thereby initiating the degradation process. It was observed that the main photodegradation products were the N-deethylated intermediates at *m*/*z* 415 [54,61,62,63] and *m*/*z* 397 (the 28 mass unit difference between *m*/*z* 443 and *m*/*z* 415 corresponded to the loss of one ethyl group, which is characteristic of the first step of N-deethylation). The subsequent transition to the ion at *m*/*z* 397 indicates an additional transformation via a decarboxylation mechanism [61]. The subsequent deethylation step, which leads to the formation of more stable products, is presumed to be responsible for the ions observed at *m*/*z* 340, which displayed a continuous increase (rising during the first 30 min and maintaining high values up to 120 min), indicating the formation of a more stable fragment resulting from the further degradation of the preceding intermediates. Lower fragments, *m*/*z* 221, likely arise as a consequence of further cleavage of the aromatic system [61]. These peaks confirm a gradual fragmentation toward smaller molecules. We hypothesize that the ions at *m*/*z* 453, *m*/*z* 459 [65], and *m*/*z* 481 are predominantly transient signals in the early stages of degradation characteristic of short-lived intermediates that rapidly transform or may represent the presence of an impurity. The changes in the MS peak intensities clearly indicate that the photodegradation of Rhodamine B follows a mechanism in which the initial rapid decrease in the parent ion (*m*/*z* 443) is accompanied by the simultaneous formation of N-deethylation intermediates (*m*/*z* 415 and *m*/*z* 397) along with a further deethylation step (*m*/*z* 340 and lower fragments). This pattern, confirmed by the temporal changes in intensity, is largely consistent with the results presented in previous studies [54,61,62,63,64,65]. Based on the current mass spectrum analysis, the possible photodegradation mechanism of RhB is proposed in Scheme 2.

#### 3.3.3. Degradation Pathway of the MB/RhB Mixture

**Figure 12:**
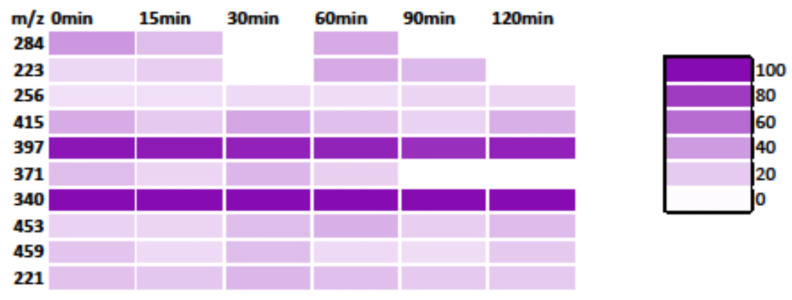
Heatmap illustrating the degradation ion profile of the MB/RhB mixture (0–120 min) in the presence of Zn_2_SO_4_/SnO_2_ under natural sunlight.

Based on the heatmap shown in Figure 12, we assumed that both dyes degraded through characteristic pathways: MB primarily via N-demethylation and the formation of “azure” intermediates, and RhB predominantly through sequential N-deethylation followed by fragmentation of the xanthene ring. More stable ions (e.g., *m*/*z* 340 for RhB and *m*/*z* 223/256 for MB) initially dominated and then gradually decreased as photodegradation proceeded toward smaller, final fragments. We further assumed that fluctuations in the intensity of ionic peaks arose from competing reactions (oxidation, deamination, decarboxylation, and ring cleavage) as well as potential interactions between the two dyes when present together in solution. Overall, these observations led us to assume that the photodegradation of the MB/RhB mixture occurred through a series of interconnected steps demethylation/deethylation, oxidative fragmentation, and ring cleavage, resulting in the gradual loss of the characteristic chromophoric structures and the formation of smaller, potentially mineralized products.

## 4. Conclusions

Solid-state synthesis was applied to obtain a Zn_2_SnO_4_/SnO_2_ nanocomposite with ~70% Zn_2_SnO_4_ and ~30% SnO_2_. Detailed structural and morphological characterization confirmed the formation of the cubic spinel Zn_2_SnO_4_ (crystallite size 108 nm) and SnO_2_ (crystallite size 88 nm) heterojunction nanocomposite powder. The estimated band gap value from the absorption edge was 3.68 eV, and the first derivative of the measured reflectance enabled the identification of additional transition energy that can be attributed to the occurrence of heterojunctions between Zn_2_SnO_4_ and SnO_2_. Analysis of the photoluminescence spectra showed emissions that can be attributed to luminescent centers due to defect energy levels originating from oxygen vacancies, tin vacancies, or oxygen interstitials. The photocatalytic activity of this nanocomposite was evaluated on single-component organic dyes RhB and MB as well as a multicomponent system containing a mixture of RhB and MB under natural sunlight irradiation. A high degradation rate of 99.1% (*k* = 0.03828 min^−1^) was achieved for MB after 120 min irradiation, while 70.6% (*k* = 0.01018 min^−1^) of RhB degraded in the same time using 50 mg of the photocatalyst material. In the case of the multicomponent system, 15 mg of the photocatalyst material achieved a degradation efficiency of 97.9% for MB. The degradation pathway was described for MB, RhB, and MB+RhB, showing that in the mixed (multicomponent) dye system, it occurred through a series of interconnected steps consisting of demethylation/deethylation, oxidative fragmentation, and ring cleavage, resulting in the gradual loss of the characteristic chromophoric structures and the formation of smaller, potentially mineralized products.

## Figures and Tables

**Table 1:** Photocatalytic degradation efficiency (DE) and pseudo-first-order kinetic constant (k) determined for different Zn_2_SnO_4_/SnO_2_ nanocomposite morphologies applied as photocatalysts for MB.

Sample	Conditions	DE (%)	*k* (min^−1^)	Ref.
Zn_2_SnO_4_/SnO_2_ nanocubes Zn:Sn = 1:1	100 mg-100 mL 2.5 ppm, UV-light	91.6% 360 min	0.007236	[23]
Zn_2_SnO_4_/SnO_2_ nanocubes Zn:Sn = 3:2	100 mg-100 mL 2.5 ppm, UV-light	99.4% 360 min	0.012244	[23]
Zn_2_SnO_4_/SnO_2_ nanoparticles	100 mg-100 mL 2 ppm UV-light	98.6% 220 min	0.01763	[19]
Zn_2_SnO_4_/SnO_2_ nanocubes	100 mg-100 mL 2 ppm UV-light	85.1% 220 min	0.008893	[19]
Zn_2_SnO_4_/SnO_2_ spheres	5 mg-50 mL 10 ppm UV light	60% 140 min	0.0049	[53]
Zn_2_SnO_4_/SnO_2_ urchin-like hollow spheres	5 mg-50 mL 10 ppm UV light	97% 140 min	0.0209	[53]
Zn_2_SnO_4_/SnO_2_ hollow cubes	100 mg-100 mL, 10 ppm sim. sunlight	97.1% 150 min	0.0241	[26]
Zn_2_SnO_4_/SnO_2_ solid cubes	100 mg-100 mL 10 ppm sim. sunlight	83.2% 150 min	0.0114	[26]
Zn_2_SnO_4_/SnO_2_ heterojunctions	50 mg-50 mL 10 ppm natural sunlight	99.1% 120 min	0.03828	This work

**Table 2:** Photocatalytic degradation efficiency (DE) and pseudo-first-order kinetic constant (k) determined for different Zn_2_SnO_4_/SnO_2_ nanocomposite morphologies applied as photocatalysts for RhB.

Sample	Conditions	DE (%)	*k* (min^−1^)	Ref.
Zn_2_SnO_4_/SnO_2_ double shell nanocubes	50 mg-50 mL 6.4 ppm, simulated sunlight	95%, 120 min	0.0214	[24]
Zn_2_SnO_4_/SnO_2_ yolk shell nanocubes	50 mg-50 mL 6.4 ppm, simulated sunlight	85%, 240 min	0.0074	[24]
Zn_2_SnO_4_/SnO_2_ hollow microbox	20 mg-50 mL 10 ppm simulated sunlight	83%, 45 min	-	[25]
Zn_2_SnO_4_/SnO_2_ nanocomposite	40 mg-100 mL 5 ppm, UV-C light	62% 60 min 94.5%, 240 min	0.0111	[22]
Zn_2_SnO_4_/SnO_2_ microspheres consisting of nanobelts	80 mg on stainless steel mesh 100 mL, 10 ppm, simulated UV–Vis light	93.4% 100 min	0.028	[55]
Zn_2_SnO_4_/SnO_2_ microspheres— nanoparticle-based nanosheets	80 mg on stainless steel mesh 100 mL, 10 ppm simulated UV–Vis light	99.9% 100 min	0.036	[55]
Zn_2_SnO_4_/SnO_2_ heterojunctions	50 mg-50 mL 10 ppm natural sunlight	70.6%, 120 min	0.0108	This work

**Table 3:** Exposure time and ion masses of the degradation products of MB and their possible structures.

Ion *m*/*z*	Exposure Time (min)	(Possible) Structure
188.17	60	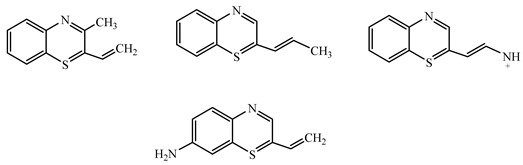
172.19	60	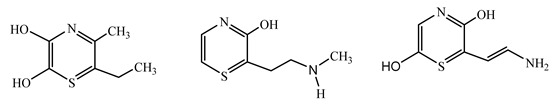
158.18	60	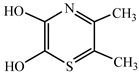
143.16	60	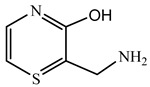
127.90	90	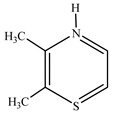
188.17	60	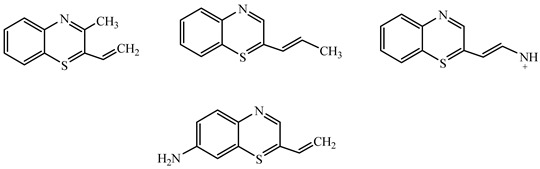
172.19	60	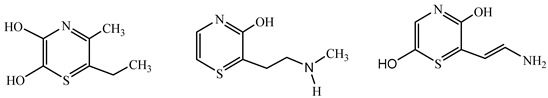

## Data Availability

Data available on request from the authors.
